# Alignment between healthy and sustainable diets: comparing the 2019 and 2025 adaptations of the planetary health diet with the healthy eating index in the Bavarian population

**DOI:** 10.3389/fnut.2026.1740957

**Published:** 2026-04-29

**Authors:** Sebastian Gimpfl, Nadine Ohlhaut, Florian Rohm, Nina Wawro, Christine Röger, Melanie Senger, Martin Kussmann, Jakob Linseisen, Kurt Gedrich

**Affiliations:** 1 ZIEL – Institute for Food and Health, AG Public Health Nutrition, Technical University of Munich, Freising-Weihenstephan, Germany; 2 Chair of Epidemiology, University of Augsburg, Augsburg, Germany; 3 Institute of Epidemiology, Helmholtz Centre Munich, Neuherberg, Germany; 4 Competence Center for Nutrition (KErn), Bavarian Research Institution for Agriculture (LfL), Freising, Germany; 5 Biometry, and Epidemiology, Medical Faculty, Institute for Medical Information Processing, Ludwig-Maximilians-University of Munich, Munich, Germany

**Keywords:** 3rd Bavarian Food Consumption Survey, Bavaria, BVS III, environmental impact, healthy eating index, planetary health diet, planetary health diet index, sustainable nutrition

## Abstract

**Introduction:**

Global food systems face the double challenge of ensuring human health and reducing environmental impacts. While the Healthy Eating Index (HEI) evaluates nutritional quality, the Planetary Health Diet (PHD) attempts to integrate ecological sustainability with healthy nutrition in its framework. This study assessed the agreement between the metric HEI-2015 (mHEI-2015) and two versions of a Planetary Health Diet Index (PHDI-2019 and its updated version PHDI-2025), as well as their associations with environmental indicators in a Bavarian population.

**Methods:**

Cross-sectional data from the 3rd Bavarian Food Consumption Survey (BVS III, 2021–2023, *n =* 1,100, 18–75 years) were used to calculate mHEI-2015, PHDI-2019, and PHDI-2025, with the latter newly operationalized to reflect the updated 2025 PHD framework. Dietary greenhouse gas emissions (GHGE), land use (LU), and water footprint (WFP) were estimated. Statistical analyses included weighted kappa (*kw*) for agreement, correlation analyses, and survey-weighted regression models adjusted for sociodemographic factors.

**Results:**

Mean mHEI-2015 was 51.3 out of 100 points, PHDI-2019 19.3 out of 42 points, and PHDI-2025 20.6 out of 45 points. Regression analyses showed that female participants, individuals with higher education, and residents of large cities (≥500,000 inhabitants) consistently scored higher across all indices (all *p* ≤ 0.047), whereas age was positively associated only with mHEI-2015 (*β* = 1.9 per 10 years; *p* < 0.001). Agreement between PHDI-2025 and PHDI-2019 was nearly perfect [weighted kappa (*kw*) = 0.84, *p* < 0.001]. Agreement between PHDI-2025 and mHEI-2015 was modest (*kw* = 0.38, 95% *CI* 0.35–0.42; *r* = 0.58; *p* < 0.001). Across PHDI-2025 quintiles, higher adherence was associated with lower GHGE (−12.5%; *p-trend* <0.001) and LU (−28.6%; *p-trend* <0.001), while WFP was unchanged (*p-trend* = 0.146). Across quintiles, the mHEI-2015 showed reduced LU (−16.3%; *p-trend* = 0.008), increased WFP (+16.7%; *p-trend* <0.001), and no GHGE change (*p-trend* = 0.849).

**Conclusion:**

Health- and sustainability-focused diet indices aligned only partially in the Bavarian population. Both PHDI versions, but not mHEI-2015, were associated with lower GHGE and LU. PHDI-2025 closely mirrored PHDI-2019, indicating refinement rather than redefinition.

## 1 Introduction

The 17 Sustainable Development Goals (SDGs) were established by the United Nations in 2015 and were accepted by all member states (1). The SDGs depict a blueprint to ensure a sustainable future, globally, by, among others, eradicating poverty, improving education and gender equality, reducing resource depletion, and steering the global food systems toward a more sustainable and healthier path (1, 2). Food systems provide an array of potentially adjustable parameters that are essential for achieving several SDGs and in turn influence or even determine the variety of foods constituting diet (3). As these food systems contribute significantly to greenhouse gas emissions (GHGE), biodiversity loss, and resource depletion (4), assessing diet quality from both nutritional and ecological perspectives is essential.

The Healthy Eating Index (HEI) and the Planetary Health Diet (PHD), two prominent food-based nutritional quality evaluators, offer insights into dietary quality. While HEI primarily evaluates the adherence to Dietary Guidelines for Americans (5) and has been linked to favorable health outcomes, including lower risk of all-cause mortality and major chronic diseases (6–8), it does not account for the ecological sustainability of dietary choices. Therefore, the EAT-Lancet Commission proposed the first iteration of the PHD in 2019 (PHD-2019), a reference diet that integrates both health and environmental considerations (9). However, the EAT-Lancet report did not mark the beginning of sustainability concerns in nutritional science. As early as 1919, Henry Sherman raised diet-related sustainability concerns (10). In 1986, Gussow and Clancy introduced the term “sustainable diets” (11). And in 2005, Cannon and Leitzmann emphasized the concept of “planetary health” in the context of their “new nutrition science project” (12). In early October 2025, the EAT-Lancet Commission adapted their PHD (PHD-2025) (13). A key novelty was the explicit inclusion of sodium (<2 g/day) as a dietary target (13), which in 2019 had been discussed as a health concern but not formally listed in the reference diet (9).

To date, no studies have evaluated adherence to the PHD-2025 due to its recent publication. Instead, many studies have developed indices reflecting the PHD-2019 (14–23). Most applied these indices to either non-representative or less recent population samples. Beneficial major health outcomes of the adherence to the PHD-2019 were supported by a multitude of these studies, e.g., lower risks of cardiovascular disease, type 2 diabetes, all-cause mortality, among other outcomes (19, 21, 23).

Benefits beyond health outcomes were brought forward in the French NutriNet-Santé cohort, where modeled diets with higher PHD-2019 scores achieved up to 75% lower GHGE and 57% lower land use (LU) (20). The EHU12/24 study provided a crucial counterpoint. Low GHGE diets were linked to poorer HEI-2010 scores in healthy young Spanish university students (24), underscoring that environmental benefits do not automatically ensure nutritional quality. Recent U. S. National Health and Nutrition Examination Survey (NHANES) analyses showed that PHD-2019 adherence reduced dietary GHGE double compared to HEI-2015 while yielding similar cardiometabolic benefits (17, 18).

These findings highlight the need for contemporary, regionally specific analyses to clarify how dietary patterns simultaneously address health and sustainability goals. In Bavaria, where traditional diets are typically rich in animal-based foods (25, 26), understanding these relationships becomes particularly urgent. This study therefore aims to examine: (1) the agreement between metric HEI-2015 (mHEI-2015), PHD-2019, and PHD-2025 scores in recent data from the 3rd Bavarian Food Consumption Survey (BVS III); (2) their respective associations with environmental indicators; and (3) sociodemographic associations and their respective relations with environmental indicators and the extent to which the indices capture overlapping or distinct dimensions of diet quality in a region where this relationship remains unquantified and may play a critically important role for regional and national public health and environmental strategies.

## 2 Methods

### 2.1 Study design and population

**Figure 1:**
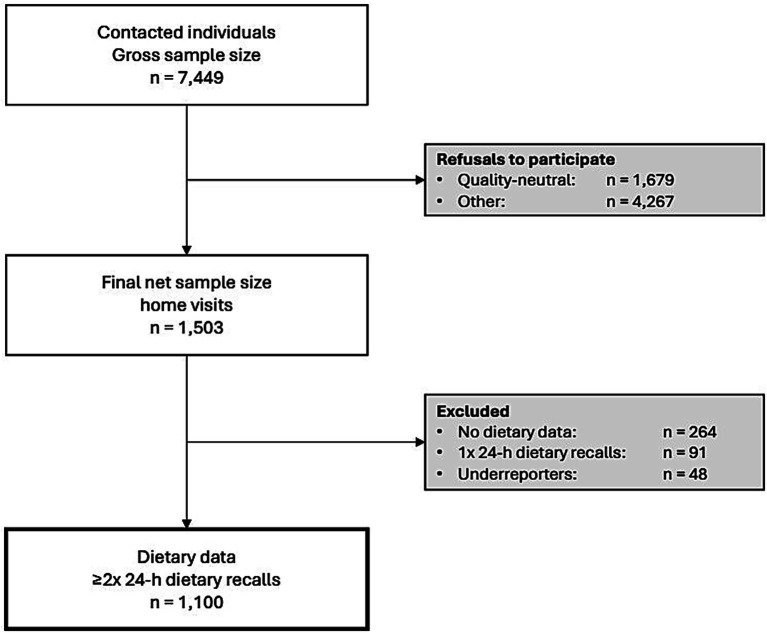
Flowchart of the 3rd bavarian food consumption study.

The BVS III was a cross-sectional, population-based survey conducted in Bavaria, Germany, between October 2021 and January 2023. A stratified, multi-stage random sampling strategy was employed to recruit individuals aged 18 to 75 years living in private households with sufficient proficiency in the German language. As the sampling frame comprised of the Bavarian adult population, no additional exclusion criteria were defined beyond stated inclusion criteria. The survey comprised two main components: a home visit and repeated telephone-based 24-h dietary recalls. During the home visit, data were collected through a combination of interviewer-led and self-reported questionnaires, waist and fasting glucose measurement, and dried blood spot collection. Full methodological details, including sampling procedures, recruitment, data collection, sampling exhaustion, and detailed reasons for non-participation, have been published previously (26, 27). The questionnaire was filled in computer-assisted. For the present analysis, only subjects with two or three 24-h dietary recalls and a usual total energy intake of at least 60% of their estimated basal metabolic rate were included (*n* = 1,100). An overview of participant flow is presented in Figure 1.

### 2.2 Dietary intake assessment

The participants’ food intake of the previous day was recorded via telephone-based 24-h dietary recalls. The recalls were conducted by trained interviewers over 6 weeks after recruitment. Participants were asked to complete ideally three non-consecutive 24-h recalls over a 6-week period, including two weekdays and one weekend day, which were randomly assigned to capture typical variation in dietary intake. These 24-h dietary recalls were carried out using GloboDiet^©^, a computer-based survey tool based on EPIC-SOFT^©^ (28), developed by the International Agency for Research on Cancer (Lyon, France). The German adaptation of the program was provided by the Max Rubner-Institute (Karlsruhe, Germany). To assist with estimating portion sizes, participants were given a 110-page photo book, featuring images of various foods and dishes in multiple portion sizes for reference. The 24-h dietary recall data were linked to the German Nutrient Database, version 3.02 (BLS 3.02) (29). Composite dishes and recipes were disaggregated into their individual ingredients based on the recipes from the BLS 3.02. The dietary data were converted into weekday-weighted averages to reflect the participants’ usual intake, accounting for variations in eating patterns between weekdays and weekends. Participants with a total energy intake-to-basal metabolic rate ratio below 0.6 were classified as extreme underreporters and excluded from dietary analyses. No overreporters were excluded as there were no participants with a total energy intake-to-basal metabolic rate ratio above 2.4. The basal metabolic rate was calculated using equations outlined by the World Health Organization (WHO) (30).

### 2.3 Dietary indices

#### 2.3.1 Planetary health diet indices

Planetary Health Diet Indices (PHDIs) were used to operationalize adherence to the PHD. In this study, two versions were computed. The first is based on the 2019 framework (PHDI-2019) and the second integrated the updates from the 2025 framework (PHDI-2025).

The PHDI-2019 (9, 31) was calculated as described by Stubbendorff et al. (23) with some adaptations. Beef, veal, lamb, pork, game (excluding game poultry), and corresponding sausage and meat products were collectively categorized as red meat as done in the 2019 EAT-Lancet Summary Report (31) and in the updated framework from 2025 (13). Saturated fat intake was included as a variable with upper intake recommendation. Intake quantities were evaluated against the PHD targets scaled to each participant’s actual energy intake. For grains, legumes, and pulses, intakes were converted to reflect the dry weight. Conversely, dried or powdered vegetables, mushrooms, fruits, and potatoes were converted into unprocessed food weight. Mushrooms were included in the vegetable group. Tree nuts, peanuts, seeds, and kernels were collectively categorized as nuts. The category fish included seafood. Grains and grain products were categorized as whole grain if the fiber content was ≥30 g per 1,000 kcal. This threshold was based on the mean fiber density of whole grain items listed in BLS 3.02. The cut-off was rounded down to the nearest multiple of 5. The group legumes included lentils, beans, peas, and soybeans. Dairy intake was measured in milk equivalents. For dairy products without additional food components, milk equivalents were calculated based on the greatest relative concentration of either calcium, essential amino acids, saturated fatty acids, or lactose compared to whole milk. For products containing other components, such as fruit yogurt, ice cream, or chocolate, only lactose content was used to determine milk equivalents. Fats and oils were classified as unsaturated if they contained ≤30% saturated fatty acids and as saturated if they exceeded 30%. The water-content in fat-reduced butter and margarines was excluded. In non-drink composite foods, the added sugar content was determined based on sucrose levels. For beverages, it was estimated using either sucrose or invert sugar content. The PHDI-2019 ranges from 0 to 42 points and comprises 14 food components, each scored from 0 to 3 points. A score of 0 represents minimal alignment with the EAT-Lancet dietary targets for that component, while a score of 3 indicates maximum alignment, as detailed in Table 1.

**Table 1:** Scoring scheme for the planetary health diet index-2019 (PHDI-2019), based on the 2019 EAT-Lancet reference diet (9) and adapted from the scoring method of Stubbendorff et al. (23), licensed under CC BY 4.0.

PHDI-2019		Intake in g/d for				
Food components	Target intake (reference range) in g	3 points	2 points	1 point	0 points	Description
Emphasized intake						
Vegetables	300 (200–600)	≥300	<300 and ≥200	<200 and ≥100	<100	*3 points:* Intake matches or exceeds the target *2 points:* Intake matches the lower limit or is between the lower limit of the reference range and the target *1 point:* Intake is 50- < 100% of the lower limit of the reference range *0 points:* Intake is less than 50% of the lower limit of the reference range
Fruits	200 (100–300)	≥200	<200 and ≥100	<100 and ≥50	<50	
Unsaturated fats^1^	40 (20–80)	≥40	<40 and ≥20	<20 and ≥10	<10	
Nuts	50 (0–100)	≥50	<50 and ≥25	<25 and ≥12.5	<12.5	*3 points:* Intake matches or exceeds the target *2 points:* Intake is 50- < 100% of the target *1 point:* Intake is 25- < 50% of the target *0 points:* Intake is less than 25% of the target
Fish	28 (0–100)	≥28	<28 and ≥14	<14 and ≥7	<7	
Whole grains	232	≥232	<232 and ≥116	<116 and ≥58	<58	
Legumes	75 (0–150)	≥75	<75 and ≥37.5	<37.5 and ≥18.75	<18.75	
Limited intake						
Red meat (beef, veal, lamb, pork, and game; excluding game poultry)	14 (0–28)	≤14	>14 and ≤28	>28 and ≤56	>56	*3 points*: Intake matches or is below the target *2 points*: Intake is between the target and the upper limit of the reference range or matches the upper limit *1 point*: Intake is >100–200% of the upper limit of the reference range *0 points*: Intake exceeds 200% of the upper limit of the reference range
Poultry (including game poultry)	29 (0–58)	≤29	>29 and ≤58	>58 and ≤116	>116	
Eggs	13 (0–25)	≤13	>13 and ≤25	>25 and ≤50	>50	
Dairy (as milk equivalents)	250 (0–500)	≤250	>250 and ≤500	>500 and ≤1,000	>1,000	
Potatoes	50 (0–100)	≤50	>50 and ≤100	>100 and ≤200	>200	
Sugars^3^	31 (0–31)	≤31	>31 and ≤62	>62 and ≤124	>124	*3 points*: Intake matches or is below the target *2 points:* Intake is >100–200% of the target *1 point:* Intake is >200–400% of the target *0 points:* Intake exceeds 400% of the target
Saturated fats^2,3^	11.8 (0–11.8)	≤11.8	>11.8 and ≤23.6	>23.6 and ≤47.2	>47.2	

PHDI-2019 Planetary Health Diet Index 2019. ^1^Saturated fatty acid proportion below 30%. ^2^Saturated fatty acid proportion equal or higher than 30%. ^3^Because the upper limits of the reference range and the target intake were identical, we set the upper bound to be twice the target intake, as described by Stubbendorff et al. (23). Both upper bounds are in line with the WHO of ≤10% of the total energy intake for a sugar intake of 62 g and a saturated fat intake of 23.6 g (69, 70). Reference intake values assume 2,500 kcal/day; in this study, targets were scaled to each participant’s actual energy intake.

For PHDI-2025, the index was computed in the same way, but intake targets were adapted to reflect the updated 2025 PHD framework (13). In contrast to PHDI-2019, sodium was introduced as an additional component. Accordingly, the PHDI-2025 ranges from 0 to 45 points and comprises 15 food components, each scored from 0 to 3 points, as detailed in Table 2.

**Table 2:** Scoring scheme for the planetary health diet index-2025 (PHDI-2025), based on the updated EAT-Lancet reference diet (13) and adapted from the scoring method of Stubbendorff et al. (23), licensed under CC BY 4.0.

PHDI-2025		Intake in g/d for				
Food components	Target intake (reference range) in g	3 points	2 points	1 point	0 points	Description
Emphasized intake						
Vegetables	300 (200–600)	≥300	<300 and ≥200	<200 and ≥100	<100	*3 points:* Intake matches or exceeds the target *2 points:* Intake matches the lower limit or is between the lower limit of the reference range and the target *1 point:* Intake is 50- < 100% of the lower limit of the reference range *0 points:* Intake is less than 50% of the lower limit of the reference range
Fruits	200 (100–300)	≥200	<200 and ≥100	<100 and ≥50	<50	
Unsaturated fats^1^	40 (20–80)	≥40	<40 and ≥20	<20 and ≥10	<10	
Nuts	50 (0–75)	≥50	<50 and ≥25	<25 and ≥12.5	<12.5	*3 points:* Intake matches or exceeds the target *2 points:* Intake is 50- < 100% of the target *1 point:* Intake is 25- < 50% of the target *0 points:* Intake is less than 25% of the target
Fish	30 (0–100)	≥30	<30 and ≥15	<15 and ≥7.5	<7.5	
Whole grains	210	≥210	<210 and ≥105	<105 and ≥52.5	<52.5	
Legumes	75 (0–150)	≥75	<75 and ≥37.5	<37.5 and ≥18.75	<18.75	
Limited intake						
Red meat (beef, veal, lamb, pork, and game; excluding game poultry)	15 (0–30)	≤15	>15 and ≤30	>30 and ≤60	>60	*3 points*: Intake matches or is below the target *2 points*: Intake is between the target and the upper limit of the reference range or matches the upper limit *1 point*: Intake is >100–200% of the upper limit of the reference range *0 points*: Intake exceeds 200% of the upper limit of the reference range
Poultry (including game poultry)	30 (0–60)	≤30	>30 and ≤60	>60 and ≤120	>120	
Eggs	15 (0–25)	≤15	>15 and ≤25	>25 and ≤50	>50	
Dairy (as milk equivalents)	250 (0–500)	≤250	>250 and ≤500	>500 and ≤1,000	>1,000	
Potatoes	50 (0–100)	≤50	>50 and ≤100	>100 and ≤200	>200	
Sugars^3^	30 (0–30)	≤30	>30 and ≤60	>60 and ≤120	>120	*3 points*: Intake matches or is below the target *2 points:* Intake is >100–200% of the target *1 point:* Intake is >200–400% of the target *0 points:* Intake exceeds 400% of the target
Saturated fats^2, 3^	11 (0–11)	≤11	>11 and ≤22	>22 and ≤44	>44	
Sodium^4^	<2	<2	≥2 and <3	≥3 and <4	≥4	*3 points*: Intake is below the target *2 points:* Intake is ≥100- < 200% of the target *1 point:* Intake is ≥200- < 400% of the target *0 points:* Intake exceeds or matches 400% of the target

PHDI-2025 Planetary Health Diet Index 2025. ^1^Saturated fatty acid proportion below 30%. ^2^Saturated fatty acid proportion equal or higher than 30%. ^3^Because the upper limits of the reference range and the target intake were identical, we set the upper bound to be twice the target intake, as described by Stubbendorff et al. (23). Both upper bounds are in line with the WHO of ≤10% of the total energy intake for a sugar intake of 60 g and a saturated fat intake of 22 g (69, 70). ^4^Unlike other components, the 2025 framework specifies only an upper limit for sodium. As both WHO (71) and Rockström et al. (13) emphasize that cardiovascular risk increases linearly above this threshold, scoring cut-offs were defined in linear increments. Reference intake values assume 2,400 kcal/day; in this study, targets were scaled to each participant’s actual energy intake.

#### 2.3.2 Healthy eating index

HEI is a widely used tool for assessing overall dietary quality, originally developed by the U. S. Department of Agriculture in 1995 and updated multiple times, with the latest version, HEI-2020, published in 2023. The HEI-2020 components and scoring standards do not differ from the previous HEI-2015. Here, the mHEI-2015 was calculated, as described by Kohl et al. (32). The mHEI-2015 ranges from 0 to 100 points and comprises 13 components, nine for adequacy and four for moderation. Scoring, as described by Kohl et al. (32) is based on energy-adjusted quantities, expressed per 1,000 kcal of intake (for most food groups) or as a percentage of energy (for fatty acids and added sugars).

### 2.4 Sustainability metrics

The dietary data were linked to two complementary sustainability metric databases. The SHARP-ID (33) was used for dietary GHGE, measured in kg CO_2_eq, and LU, measured in m^2^*yr. This database incorporates complete life cycle assessment data from four European countries (Denmark, Czech Republic, Italy, and France), with system boundaries encompassing agricultural production, processing, packaging, transportation, and participant-level preparation (33), including participants’ reported cooking methods assessed within the BVS III. SuEatableLife database (34) was, in turn, used for the WFP, measured in kiloliters. This database’s system boundaries differed by terminating at the distribution center, thus excluding post-retail stages and consequently not incorporating participants’ cooking methods. The WFP calculations focused exclusively on impacts occurring during primary production, processing, packaging, and transport to distribution centers. Because WFP is based on different system boundaries, it was interpreted as a complementary environmental indicator rather than as directly equivalent to GHGE and LU. Both databases have been peer-reviewed, and their full methodological frameworks are detailed in respective publications (33, 34).

### 2.5 Participant characteristics

Data on sex, age, body height and mass, education level, net household income, municipality category, physical activity, and smoking were collected during the home visit. Physical activity groups were classified according to Gerrior et al. (35). The equivalized net income was calculated using the modified OECD (Organization for Economic Co-operation and Development) equivalence scale (36, 37). Data on the net household income were self-reported based on predefined ranges, with the midpoint of each range used for calculations. The highest monthly income category, “7,000 Euro and above,” was interpreted as 7,500 Euro/month, while the lowest, “below 500 Euro,” was approximated as 250 Euro/month. The Body Mass Index (BMI) was calculated via self-reported weight and height. BMI categories were defined according to the WHO (38).

### 2.6 Covariates

For regression analyses, the covariates sex, age, BMI, education, equivalized net household income, and municipality category were included to account for relevant participant characteristics and potential confounding in the associations between sociodemographic and anthropometric factors and dietary indices.

### 2.7 Weighting

To ensure the data were representative of the Bavarian population, weighting factors were applied to the data to adjust for administrative district, education level, and combinations of education with age, as well as gender with age. The reference population was based on the German 2020 microcensus (39), i.e., an extrapolation for the Bavarian population. Weighting factors were calculated based on three steps. First, design weighting was used to correct for unequal selection probabilities arising from the sample-point distribution. Second, non-response weighting was applied based on model-based estimates of response and participation probabilities derived from the gross sample. And third, the net sample was calibrated to Bavarian population margins from the microcensus using an iterative raking procedure. For dietary data, an additional weighting step was applied to account for the reduced subsample. The present analyses were conducted using the final weights and design-based variance estimation without cluster or strata variables.

In addition, dietary data were weighted by weekday to correct for the unequal distribution of survey days, ensuring that weekdays and weekends were proportionally represented in the analysis.

### 2.8 Statistical analysis

Descriptive statistics were used to summarize sociodemographic characteristics of the study population, using standard deviation (SD) for unweighted and standard error (SE) for weighted data. Wilcoxon’s and Kruskal-Wallis rank sum tests were used to assess group differences. Associations between dietary indices and sociodemographic characteristics were examined using survey-weighted linear regression, adjusting for sex, age, BMI, education, equivalized net income, and municipality category. Regression assumptions (linearity, homoscedasticity, and normality of residuals) were assessed graphically. Multicollinearity was evaluated using variance inflation factors with cut-offs of <2. Design-based Pearson’s correlation coefficients were calculated to assess the linear associations between the overall scores and components of the PHDIs and mHEI-2015. Correlation strength was interpreted according to Schober et al. (40). Weighted kappa (*kw*) statistics with linear weights were calculated (41) to assess the agreement between the PHDIs and mHEI-2015 with interpretation based on Fleiss et al. (42). Participants were categorized into quintiles of adherence (“lowest” to “highest”) for kappa analyses and for evaluating dietary environmental impacts. For component-level agreement, participants were grouped into tertiles (“low” to “high”). Linear trend analyses for environmental impacts across quintiles were performed using survey-weighted generalized linear models with ordered factors. In addition, survey-weighted generalized linear models with the indices entered as standardized continuous variables were used to estimate effects on environmental metrics, adjusted to 2,500 kcal, per SD increase of the respective index. Apart from descriptive statistics of the unweighted study sample, all tests accounted for survey adjustments to represent the Bavarian population. Weighted descriptive statistics and regression models were estimated using survey-weighted procedures implemented in the R packages survey (43) and srvyr (44). Variance estimation was performed using design-based standard errors based on Taylor series linearization (43). Linear regression models, correlations, and generalized linear models for trend analyses were all conducted within this survey-weighted framework. Missing data occurred for the variable equivalized net household income. In analyses using equivalized net household income as covariate, participants with missing values had to be excluded listwise, resulting in a reduced sample size (weighted *n* = 1,018). All other analyses were conducted using the full sample. Statistical analyses and graphical depictions were performed using the statistical software R version 4.4.0 (45).

## 3 Results

### 3.1 Study population

The study population consisted of 1,100 individuals, who completed at least two 24-h dietary recalls. In the weighted study population, the distribution between males and females was nearly equal (males: 51%; females: 49%). The mean age was 46.7 (*SE*: 0.8) years and the mean BMI 26.0 (*SE*: 0.3) kg/m^2^. More than half were overweight (51%) (see Table 3), i.e., either pre-obese (BMI ≥ 25 to <30 kg/m^2^) or obese (BMI ≥ 30 kg/m^2^). The characteristics of the unweighted study population are shown in Supplementary Table 1.

**Table 3:** Sociodemographic characteristics, planetary health diet index-2019, planetary health diet index-2025, and mHEI-2015 of the weighted study population (*n* = 1,100).

Variable	*n* = 1,100
Sex	
Male	51 (558)
Female	49 (542)
Mean (SE) age in years	46.7 (0.8)
Age group	
18–24	10 (105)
25–34	18 (202)
35–50	28 (311)
51–64	29 (317)
≥65	15 (165)
Mean (SE) BMI in kg/m^2^	26.0 (0.3)
BMI group	
Underweight	2 (18)
Normal weight	47 (514)
Pre-obesity	33 (362)
Obesity	19 (206)
Education	
Low	33 (365)
Medium	29 (322)
High	38 (413)
Smoking status	
Never	50 (554)
Currently	17 (184)
In the past	33 (362)
Physical activity group	
Sedentary	23 (258)
Low active	24 (261)
Variable	*n* = 1,100
Active	23 (256)
Very active	30 (326)
Mean (median; IQR) planetary health diet Index-2019	19.3 (19.0; 16.0, 22.0)
Mean (median; IQR) planetary health diet index-2025	20.6 (20.0; 17.0, 23.0)
Mean (median; IQR) mHEI-2015	51.3 (50.5; 42.8, 60.0)

BMI, body mass index; IQR, interquartile range; SE, standard error. Values are given as % (n), unless otherwise specified.

### 3.2 Indices

**Figure 2:**
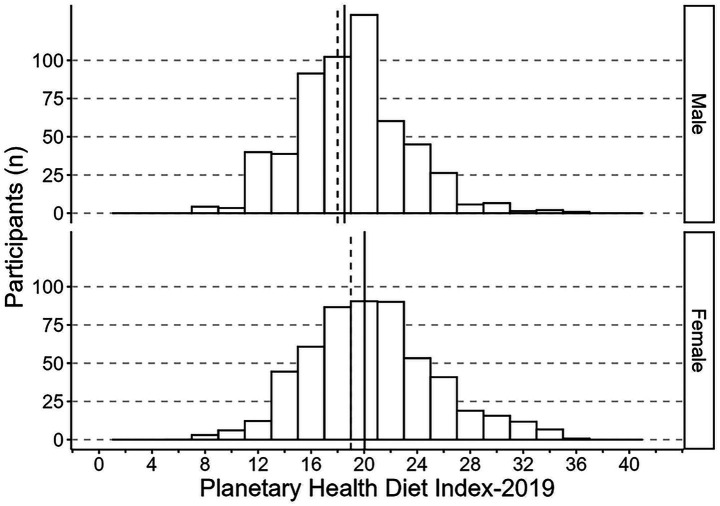
Histogram of the Planetary Health Diet Index-2019 distribution (range: 0–42 points) among 558 males and 542 females in the 3rd Bavarian Food Consumption Study. The continuous lines depict the group mean and dashed the median. Data are weighted to represent the Bavarian population.

**Figure 3:**
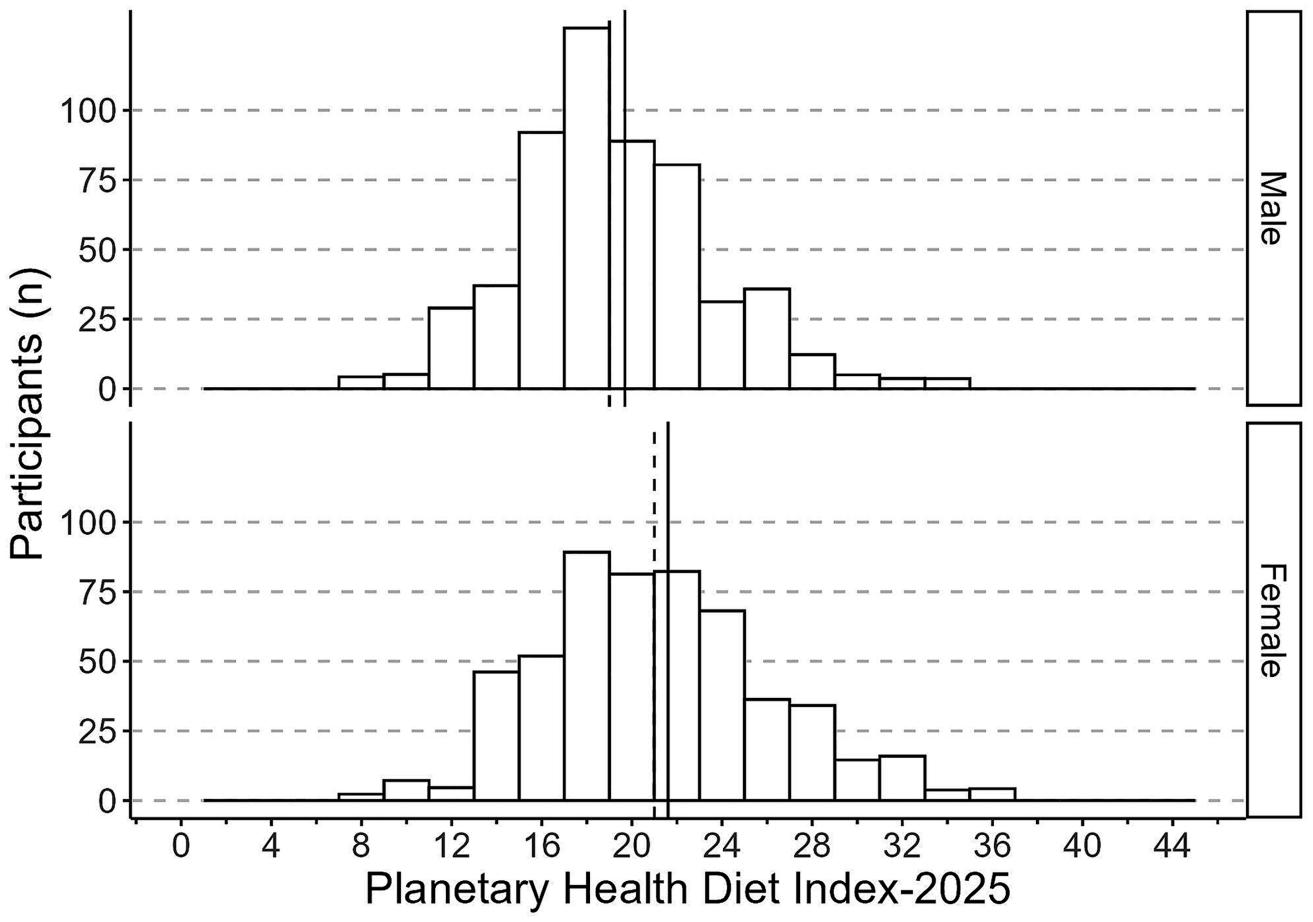
Histogram of the Planetary Health Diet Index-2025 distribution (range: 0–45 points) among 558 males and 542 females in the 3rd Bavarian Food Consumption Study. The continuous lines depict the group mean and dashed the median. Data are weighted to represent the Bavarian population.

**Figure 4:**
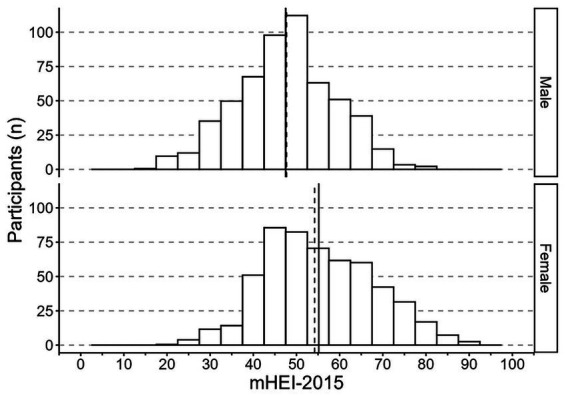
Histogram of the mHEI-2015 distribution (range: 0–100 points) among 558 males and 542 females in the 3rd Bavarian Food Consumption Study. The continuous lines depict the group mean and dashed the median. Data are weighted to represent the Bavarian population. mHEI metric Healthy Eating Index.

The mean (*M*) PHDI-2019 was 19.3 (*SE* = 0.2), corresponding to 46.0% of the maximum 42 points, with a median of 19.0. The participants scored a minimum of 6 and a maximum of 35 points. Women scored on average higher (*M* = 20.0, *SE* = 0.3) than men (*M* = 18.5, *SE* = 0.3; *p* = 0.001) (Figure 2). For the updated PHDI-2025, the mean score was 20.6 (SE = 0.2), equivalent to 45.8% of the maximum 45 points, with a median of 20.0. Values ranged from 8 to 37. Women again scored higher (*M* = 21.6, *SE* = 0.3) than men (*M* = 19.7, *SE* = 0.3; *p* < 0.001) (Figure 3). Regarding the mHEI-2015, the participants scored between 16.5 and 90.4 out of 100 possible points. The mean mHEI-2015 was 51.3 (*SE* = 0.6, 51.3% adherence). Again, the median was slightly lower with 50.5 points and women showed on average a higher adherence to the mHEI-2015 (*M* = 55.2, *SE* = 0.9) than men (*M* = 47.5, *SE* = 0.8, *p* < 0.001) (Figure 4).

For all three regression models with mHEI-2015, PHDI-2019, and PHDI-2025 as dependent variables, residual diagnostics indicated no major violations of model assumptions. All variance inflation factors were below 2, suggesting no multicollinearity. To facilitate comparison across indices with different score ranges (0–42 for PHDI-2019, 0–45 for PHDI-2025, and 0–100 for mHEI-2015), regression coefficients are also expressed as percentages of the respective maximum possible score.

Model fits were modest, with adjusted *R^2^* values of 0.08 for PHDI-2019, 0.10 for PHDI-2025, and 0.14 for mHEI-2015 (all models *p* < 0.001) (Table 4). For both the PHDI-2019 and PHDI-2025, results were similar. Compared to men, women had significantly higher scores [PHDI-2019: *β* = 1.4 (3.3%); *p* = 0.002; PHDI-2025: *β* = 1.8 (4.0%); *p* < 0.001]. Compared to those with low education, participants with the highest education also had higher adherence estimates [PHDI-2019: *β* = 1.0 (2.4%); *p* = 0.047; PHDI-2025: *β* = 1.2 (2.7%); *p* = 0.030]. Finally, compared to residents of the smallest municipalities (<2,000 inhabitants), those in large cities with ≥500,000 inhabitants scored higher as well [PHDI-2019: *β* = 2.2 (5.2%); *p* = 0.010; PHDI-2025: *β* = 2.5 (5.6%); *p* = 0.005]. No significant associations were observed for age, BMI, medium education, or income in either model. For the mHEI-2015, broadly similar sociodemographic patterns were observed, with higher scores among women (*β* = 7.0, *p* < 0.001), and among participants with both medium (*β* = 4.1; *p* = 0.016) and high education (*β* = 4.7; *p* = 0.002) compared to those with low education. Adherence estimates were also higher among residents of large cities with ≥500,000 inhabitants compared to the smallest municipalities (*β* = 5.2; *p* = 0.013). In contrast to the PHDI models, mHEI-2015 was positively associated with both medium and high education. Moreover, only mHEI-2015 showed a significant age association, with scores increasing at older ages (*β* = 1.9 per 10 years; *p* < 0.001). The findings indicate that female sex, high education, and residence in large cities were consistently linked to higher diet quality across indices. Notably, only the mHEI-2015 captured a clear age effect. Full regression estimates are presented in Table 4.

**Table 4:** Multivariate linear regression estimates for associations between sociodemographic determinants and dietary quality indices (PHDI-2019, PHDI-2025, and mHEI-2015).

	PHDI-2019^1^ (scale 0–42)		PHDI-2025^2^ (scale 0–45)		mHEI-2015^3^ (scale 0–100)	
Predictor	*β* (*SE*; *95% CI*)	Statistics	*β* (*SE*; *95% CI*)	Statistics	*β* (*SE*; *95% CI*)	Statistics
Intercept	18.3 (1.9; 15.0, 22.6)	*t* = 9.7; *p* < 0.001	19.9 (2.1; 15.6, 24.1)	*t* = 9.3; *p* < 0.001	36.5 (5.7; 25.3, 47.7)	*t* = 6.4; *p* < 0.001
Sex						
Male (reference)						
Female	1.4 (0.5; 0.6, 2.3)	*t* = 3.2; *p* = 0.002	1.8 (0.5; 0.9, 2.8)	*t* = 3.9; *p* < 0.001	7.0 (1.4; 4.4, 9.7)	*t* = 5.2; *p* < 0.001
Age (per 10 years)	−0.1 (0.2; −0.4, 0.2)	*t* = −0.7; *p* = 0.471	−0.0 (0.2; −0.4, 0.3)	*t* = −0.2; *p* = 0.852	1.9 (0.4; 1.0, 2.7)	*t* = 4.4; *p* < 0.001
BMI (per 5 kg/m^2^)	−0.3 (0.3; −0.9, 0.3)	*t* = −0.8; *p* = 0.397	−0.3 (0.4; −1.0, 0.4)	*t* = −0.9; *p* = 0.375	−0.5 (1.0; −2.4, 1.4)	*t* = −0.5; *p* = 0.616
Equivalized net income (per €1,000)	0.2 (0.2; −0.2, 0.5)	*t* = 1.0; *p* = 0.313	0.2 (0.2; −0.2, 0.5)	*t* = 1.0; *p* = 0.342	0.2 (0.5; −0.7, 1.1)	*t* = 0.4; *p* = 0.723
Education						
Low (reference)						
Medium	0.2 (0.5; −0.8, 1.3)	*t* = 0.4; *p* = 0.678	0.3 (0.5; −0.8, 1.4)	*t* = 0.5; *p* = 0.594	4.1 (1.7; 0.8, 7.5)	*t* = 2.4; *p* = 0.016
High	1.0 (0.5; 0.0, 2.0)	*t* = 2.0; *p* = 0.047	1.2 (0.5; 0.1, 2.2)	*t* = 2.2; *p* = 0.030	4.7 (1.5; 1.7, 7.6)	*t* = 3.1; *p* = 0.002
Municipality category						
<2,000 inhabitants (reference)						
2,000- < 5,000 inhabitants	0.5 (0.8; −1.0, 2.0)	*t* = 0.7; *p* = 0.494	0.5 (0.8; −1.0, 2.0)	*t* = 0.6; *p* = 0.517	0.8 (1.8; −2.8, 4.4)	*t* = 0.4; *p* = 0.674
5,000- < 20,000 inhabitants	0.5 (0.7; −0.8, 1.8)	*t* = 0.7; *p* = 0.473	0.4 (0.7; −1.0, 1.7)	*t* = 0.5; *p* = 0.594	2.1 (1.8; −1.5, 5.7)	*t* = 1.2; *p* = 0.245
20,000- < 50,000 inhabitants	1.2 (0.7; −0.2, 2.5)	*t* = 1.7; *p* = 0.094	1.2 (0.7; −0.1, 2.6)	*t* = 1.8; *p* = 0.078	2.2 (1.9; −1.5, 5.9)	*t* = 1.2; *p* = 0.239
50,000- < 100,000 inhabitants	−0.4 (1.0; −2.3, 1.5)	*t* = −0.4; *p* = 0.707	−0.2 (0.9; −2.0, 1.7)	*t* = −0.2; *p* = 0.854	−2.1 (2.4; −6.8, 2.6)	*t* = −0.9; *p* = 0.382
100,000- < 500,000 inhabitants	1.4 (0.8; −0.0, 2.9)	*t* = 1.9; *p* = 0.054	1.2 (0.8; −0.3, 2.6)	*t* = 1.5; *p* = 0.130	0.4 (1.8; −3.1, 3.8)	*t* = 0.2; *p* = 0.843
≥500,000 inhabitants	2.2 (0.9; 0.5, 3.9)	*t* = 2.6; *p* = 0.010	2.5 (0.9; 0.8, 4.3)	*t* = 2.8; *p* = 0.005	5.2 (2.1; 1.1, 9.3)	*t* = 2.5; *p* = 0.013

BMI, body mass index; CI, confidence interval; mHEI-2015, metric Healthy Eating Index; PHDI-2019, Planetary Health Diet Index 2019, PHDI-2025, Planetary Health Diet Index 2025; SE, standard error. ^1^*R*^2^ = 0.09, *adj. R*^2^ = 0.08, *F*(12, 1,008) = 8.30, *p* < 0.001. ^2^*R*^2^ = 0.11, *adj. R*^2^ = 0.10, *F*(12, 1,008) = 10.36, *p* < 0.001. ^3^*R*^2^ = 0.15, *adj. R*^2^ = 0.14, *F*(12, 1,008) = 15.24, *p* < 0.001. Data are weighted to represent the Bavarian population (*n* = 1,018).

### 3.3 Agreement

Both PHDI-2019 and PHDI-2025 were found to be, albeit moderately, positively correlated with the mHEI-2015 [*r*(1,098) = 0.52 and 0.58, respectively; both *p* < 0.001] (Supplementary Figures 1, 2). The two PHDI versions were themselves highly correlated [*r*(1,098) = 0.97, *p* < 0.001] (Supplementary Figure 3). Weighted kappa statistics were calculated to evaluate the agreement between the PHDIs and mHEI-2015. There was a statistically significant but only fair agreement between the PHDI-2019 and mHEI-2015 (*kw* = 0.34, *95% CI* = 0.30–0.37, *p* < 0.001) and between the PHDI-2025 and mHEI-2015 (*kw* = 0.38, *95% CI* = 0.35–0.42, *p* < 0.001). Agreement between the two PHDI versions was almost perfect (*kw* = 0.84, *95% CI* = 0.83–0.86, *p* < 0.001).

**Figure 5:**
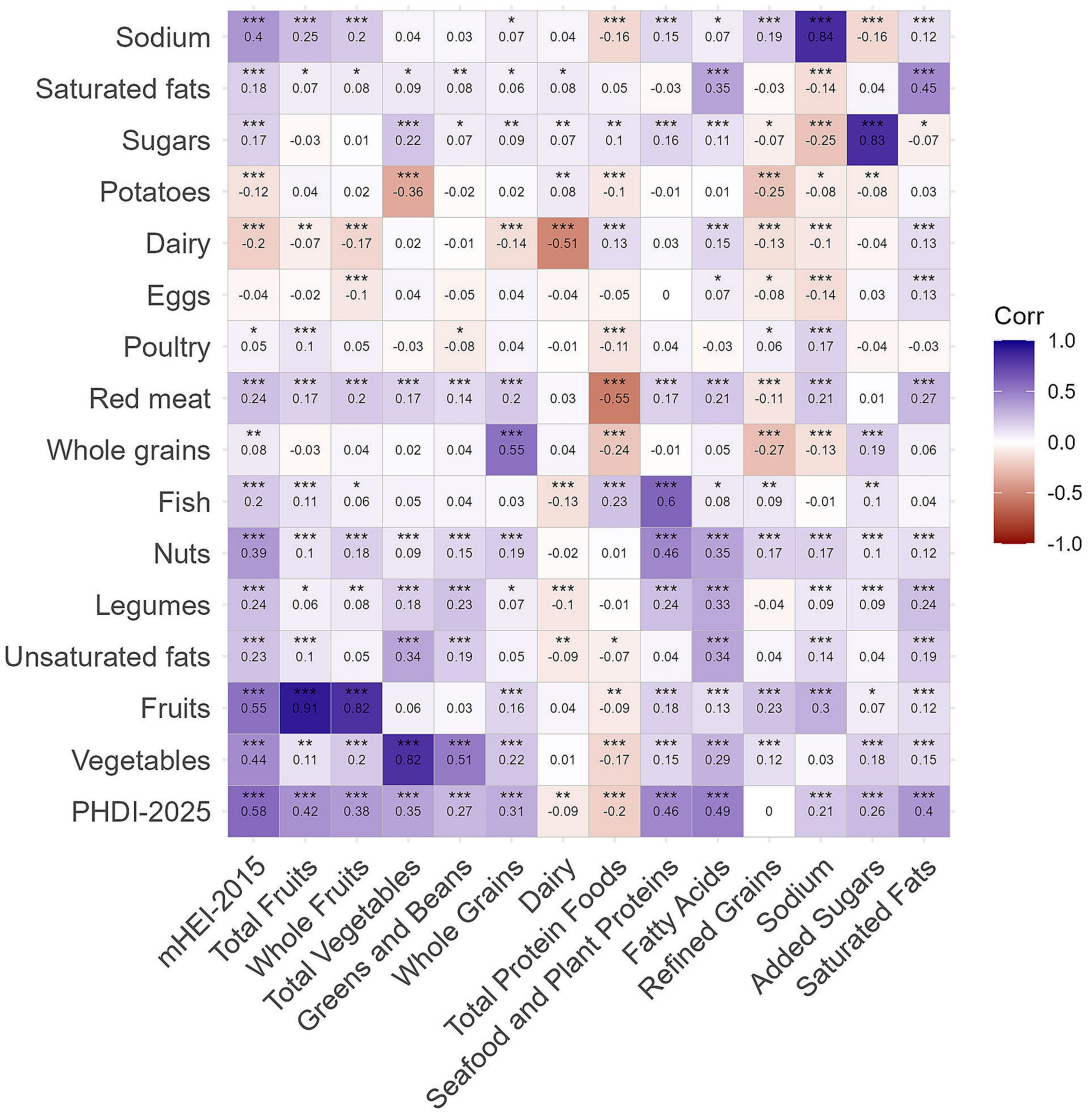
Pearson’s correlations between the Planetary Health Diet Index-2025, including its components (*y*-axis), and mHEI-2015 and its components (*x*-axis) (*n* = 1,100). Red indicates negative and blue positive correlation. No correlation is represented as white. Data are weighted to represent the Bavarian population. Significance levels: *p < 0.05, **p < 0.01, ***p < 0.001. mHEI metric Healthy Eating Index, PHDI Planetary Health Diet Index.

An accompanying correlation analysis of the individual components of PHDI-2025 and mHEI-2015 was performed to illustrate differences in their scoring schemes. Strong positive correlations were observed for comparable components, including fruits, vegetables, sugars, and sodium [all *r*(1,098) ≥ 0.82, *p* < 0.001]. Negative correlations indicated opposite valuations, e.g., between “Total Protein Foods” and “Red Meat” [*r*(1,098) = −0.55, *p* < 0.001] or between both dairy groups [*r*(1098) = −0.51, *p* < 0.001] (Figure 5). Correlation patterns were highly similar when comparing mHEI-2015 with PHDI-2019 (Supplementary Figure 4), and nearly perfect agreement was observed between identical components of both PHDI versions (Supplementary Figure 5). The newly added sodium component in PHDI-2025 showed only weak correlations with other categories, suggesting limited overlap with existing scoring dimensions.

**Figure 6:**
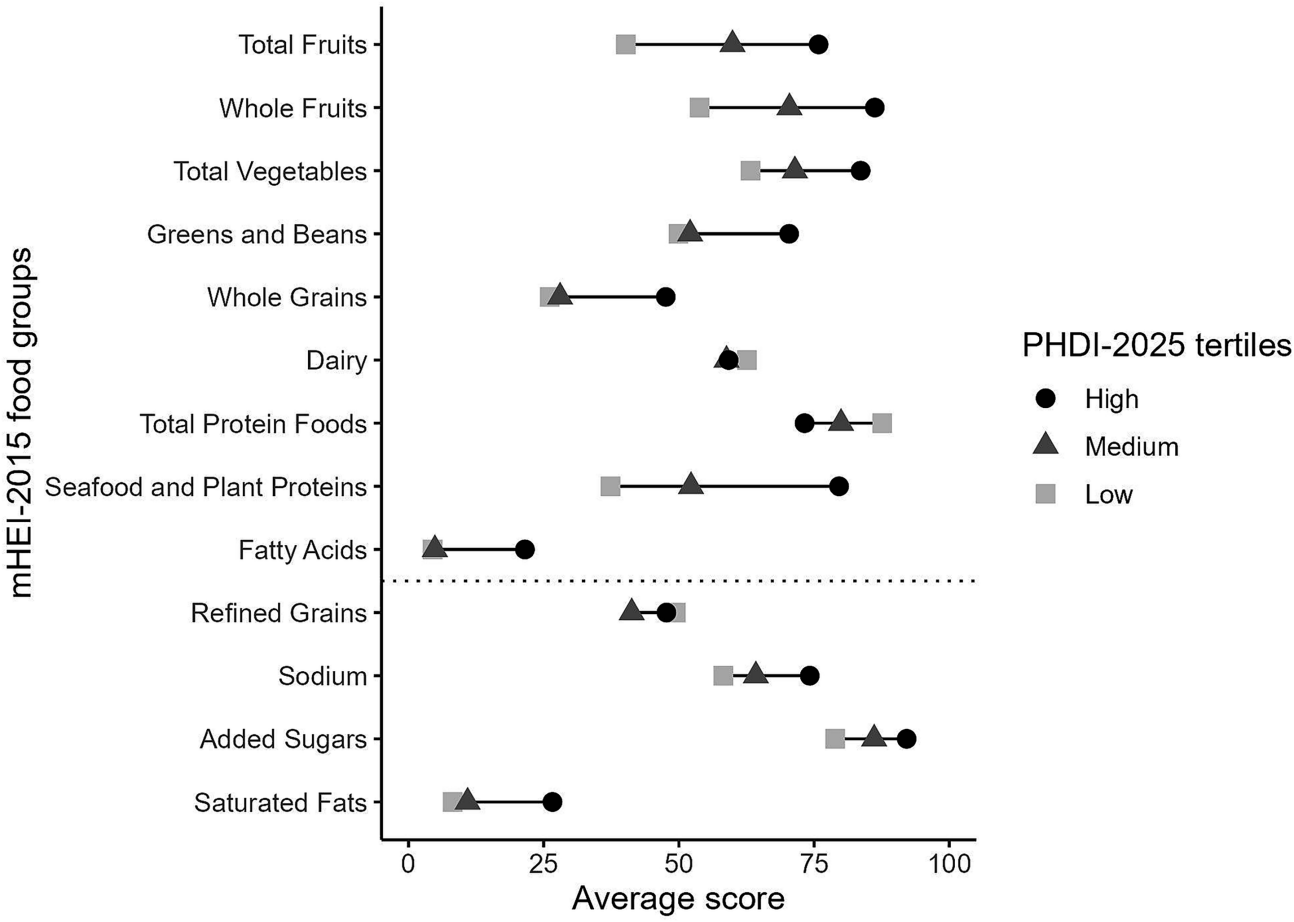
Dotchart of the average mHEI-2015 scores across food groups by Planetary Health Diet Index-2025 tertiles (*n* = 1,100). Food groups for adequacy are shown above and for moderation below the dotted line. Data are weighted to represent the Bavarian population. mHEI, metric Healthy Eating Index; PHDI, Planetary Health Diet Index.

Figure 6 shows the mean mHEI-2015 scores by PHDI-2025 tertiles differentiated by the mHEI-2015 food groups. Across PHDI-2025 tertiles, mean mHEI-2015 scores increased for plant-based food groups such as “Total Fruits,” “Whole Fruits,” “Total Vegetables,” “Greens and Beans,” “Whole Grains,” and “Seafood and Plant Proteins” (all *p-trend* <0.001). In contrast, “Total Protein Foods” scores declined (*p-trend* <0.001), reflecting the PHDI’s lower emphasis on animal-based protein. “Dairy” scores remained unchanged (*p-trend* = 0.339). “Sodium” and “Fatty Acids” showed only weak associations. Comparable patterns were observed when examining PHDI-2025 food groups across mHEI-2015 tertiles (see Supplementary Figure 6).

### 3.4 Dietary environmental impacts

**Figure 7:**
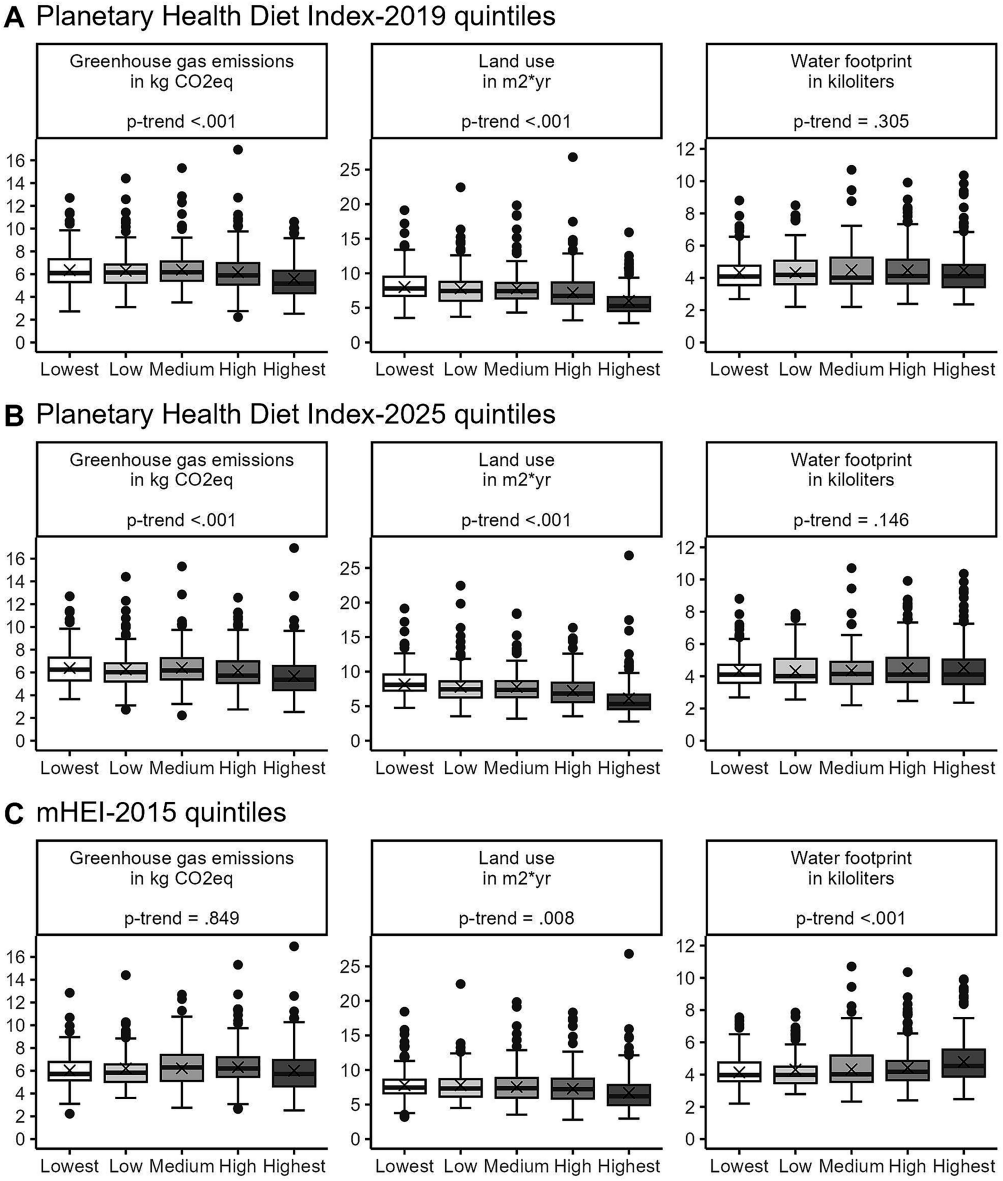
Diet-related greenhouse gas emissions, land use, and water footprint across quintiles of **(A)** Planetary Health Diet Index-2019, **(B)** Planetary Health Diet Index-2025, and **(C)** mHEI-2015 (*n* = 1,100). Data are weighted to represent the Bavarian population. WFP was estimated using a database with system boundaries different from those used for GHGE and LU; direct comparison across indicators should therefore be interpreted cautiously. CO_2_eq carbon dioxide equivalents, mHEI metric Healthy Eating Index.

Figure 7 illustrates dietary environmental impact, i.e., GHGE, LU, and WFP, across quintiles of PHDI-2019, PHDI-2025, and mHEI-2015, respectively. Environmental impact estimates were energy-adjusted and expressed per 2,500 kcal of dietary intake.

Significant negative trends were observed for GHGE and LU across PHDI-2019 quintiles (both *p-trend* < 0.001), with mean GHGE decreasing from 6.4 kg CO_2_eq (*SE* = 0.1) in the lowest quintile to 5.4 kg CO_2_eq (*SE* = 0.2) in the highest, and LU from 8.2 m^2^*yr. (*SE* = 0.2) to 5.8 m^2^*yr. (*SE* = 0.2) (Figure 7A). WFP showed no consistent pattern (*p-trend* = 0.305). Consistent results were observed in continuous models, where a SD increase in PHDI-2019 was associated with lower GHGE (*β* = −0.28 kg CO_2_eq, *p* < 0.001) and lower LU (*β* = −0.79 m^2^*yr., *p* < 0.001), while no significant association was observed for WFP (*p* = 0.139). For PHDI-2025 quintiles, results were quite similar to PHDI-2019, i.e., GHGE and LU (both *p-trend* <0.001) both decreased from the lowest to the highest quintile [GHGE: 6.4 (*SE* = 0.1) to 5.6 kg CO_2_eq (*SE* = 0.2); LU: 8.4 (*SE* = 0.2) to 6.0 m^2^*yr. (*SE* = 0.2)], while WFP remained unchanged (*p-trend* = 0.146) (Figure 7B). A one SD increase in PHDI-2025 was likewise associated with lower GHGE (*β* = −0.29 kg CO_2_eq; *p* < 0.001) and lower LU (*β* = −0.86 m^2^*yr.; *p* < 0.001), whereas no significant association was observed for WFP (*p = 0*.115). In contrast, mHEI-2015 quintiles showed a different pattern (Figure 7C). GHGE did not differ significantly across quintiles (*p-trend* = 0.849; *p* = 0.063). LU decreased with higher quintiles (*p-trend* = 0.008), ranging from 8.0 m^2^*yr. (*SE* = 0.3) in the lowest quintile to 6.7 m^2^*yr. (*SE* = 0.3) in the highest. WFP was positively associated with mHEI-2015 (*p-trend* = 0.001), with mean values increasing from 4.2 kiloliters (*SE* = 0.1) in the 1st quintile to 4.9 kiloliters (*SE* = 0.2) in the 5th quintile. Supporting this, a one SD increase in mHEI-2015 was not associated with GHGE (*p* = 0.898) but was associated with lower LU (*β* = −0.40 m^2^*yr.; *p* < 0.001) and higher WFP (*β* = 0.28 kiloliters; *p* < 0.001). The quintile-based values are presented in Supplementary Table 2.

## 4 Discussion

This study evaluated the alignment between PHDI-2019, PHDI-2025, and mHEI-2015, as well as their sociodemographic predictors and environmental impacts, using data from the BVS III. Both PHDI versions and the mHEI-2015 showed moderate average adherence, yet their associations with participant characteristics and sustainability indicators differed. Women, participants with higher education, and individuals living in large cities (≥500,000 inhabitants) consistently achieved higher scores across all indices. However, age was only positively associated with mHEI-2015, whereas no age effect was observed for either PHDI version. Agreement between the indices was statistically significant but limited in strength, suggesting that adherence to the scores may capture partly overlapping yet distinct aspects of diet quality. As expected, the two PHDI versions showed almost perfect agreement, confirming methodological consistency between the 2019 and 2025 frameworks. Higher PHDI-2019 and PHDI-2025 scores were both associated with lower GHGE and LU, while no consistent association was found with WFP. The trends across quintiles were very similar for both PHDI versions, suggesting that the adaptation in PHDI-2025, i.e., the inclusion of the sodium component and adjustment of existing categories, had little impact on overall score or environmental associations. In contrast, mHEI-2015 showed no significant association with GHGE, a weak inverse association with LU, and a positive association with WFP.

The Food and Agriculture Organization of the United Nations (FAO) defines sustainable diets as “those diets with low environmental impacts which contribute to food and nutrition security and to healthy life for present and future generations. Sustainable diets are protective and respectful of biodiversity and ecosystems, culturally acceptable, accessible, economically fair and affordable; nutritionally adequate, safe and healthy; while optimizing natural and human resources” (46). Accordingly, sustainability in nutrition research can be approached from several distinct but complementary perspectives. Straightforwardly, environmental sustainability was assessed empirically through environmental indicators derived from, i.e., LCA models, such as GHGE, LU, and WFP, as done in present work. At the same time, sustainability may also be operationalized through normative dietary frameworks such as the PHD. The PHD includes predefined dietary targets intended to balance human health and planetary boundaries (9, 13). Dietary environmental metrics inherently capture only a part of dietary sustainability whereas multidimensional frameworks capture a broader sustainability concept. It is important to clarify this distinction. Empirical variables, such as GHGE, LU, and WFP, represent only one dimension of sustainable diets within a broader multidimensional framework. Similarly, the HEI represents a normative dietary framework that captures predefined aspects of a healthy dietary pattern based on the Dietary Guidelines for Americans (32). As with the PHD, the HEI therefore reflects a specific conceptualization of diet quality.

### 4.1 Study conduction

Contemporary population research faces immense challenges in achieving representative participation. The response rate of the BVS III amounts to 26.0% (27) exemplifying two distinct dimensions of these challenges: a well-documented decline in survey participation over the last decades predating the COVID-19 pandemic, and additional pandemic-specific factors, as we established in prior publications (26, 27). Dietary intake in BVS III was assessed using repeated 24-h dietary recalls, with at least two non-consecutive recall days required for inclusion. This approach follows recommendations of the European Food Safety Authority for national dietary surveys, which state that at least two days are sufficient to estimate usual dietary intake (47). The same methodological approach has also been applied in previous German national dietary surveys, including the 2nd National Nutrition Survey (NVS II) (48), ensuring comparability across studies. However, there is ongoing discussion in the literature regarding the optimal number of dietary assessment days, with some studies suggesting that three or more days may further improve the precision of usual intake estimates for certain nutrients (49, 50). Missing data occurred for the variable equivalized net household income. In the regression analyses using equivalized net household income as covariate, participants with missing values were excluded listwise, resulting in a reduced sample size. To evaluate the potential impact of missing income data, the regression models were repeated without inclusion of equivalized net household income as a covariate. The results remained materially unchanged, indicating that the main findings were robust to the handling of missing income data (data not shown).

### 4.2 mHEI-2015

For the evaluation of the overall diet quality, the mHEI-2015 was applied. This metric version of the HEI is conceptually and structurally equivalent to the most recent version, the HEI-2020, which introduced no changes beyond updated nomenclature to clarify that it aligned with the most recent 2020–2025 Dietary Guidelines for Americans. The mHEI-2015, as described by Kohl et al. (32), was developed to adjust the HEI-2015 to be operational with metric units and European food composition databases. Our focus was not on adherence to past or current national targets, but rather on evaluating general patterns of health-promoting eating behavior and their alignment with PHDI. The mHEI-2015 offers a conceptually coherent and internationally comparable framework for assessing diet quality since it has been consistently linked to favorable health outcomes (6). Nonetheless, HEI has recognized limitations. As a guideline-based index, it is inherently dependent on the Dietary Guidelines for Americans, which requires specific data structures for its application (51). Due to this, its transferability to non-U. S./non-Western is limited. Dietary patterns in culturally different or multicultural settings may not be adequately captured despite being health-promoting (52). As of this, mHEI-2015 was used as established and widely used tool to approximate diet quality, while acknowledging that it reflects selected aspects of healthy eating. In the present study, participants achieved an average mHEI-2015 score of 51.3 points on the scale ranging from 0 to 100. Comparable values were reported in U. S. adults from NHANES 2017–2018 (*M* = 52.6) and NHANES 2015–2018 (*M* = 53.0) (18, 32), as well as in a young Turkish university cohort (*M* = 54.2) (53). Overall, the mHEI-2015 scores in the Bavarian population are comparable to those reported in other high-income countries, indicating similarly suboptimal dietary quality (18, 22, 32, 53, 54).

### 4.3 PHDI

Unlike HEI, which is a well-established index to evaluate diet quality, there is currently no universally standardized method for quantifying adherence to either the 2019 or 2025 version of the PHD. Accordingly, multiple measurement approaches have been proposed differing in aspects such as included components, scoring systems, levels of complexity, and score ranges (15, 16, 20, 21, 23). Inspired by Cacau et al. (15), we adapted the method initially developed by Stubbendorff et al. (23) and categorized the food components as either “emphasized” or “limited” foods. Scoring is done on a scale of 0–3 for “emphasized foods” and inversely for “limited foods,” resulting in a total score from 0 to 42 points for PHDI-2019 (23) and from 0 to 45 for PHDI-2025 allowing for a nuanced assessment without being excessively complex. In contrast to Stubbendorff et al. (23), we combined beef, veal, lamb, pork, and game into a unified “red meat” category, as done in the simplified version of the PHD-2019 in the summary report of the EAT-Lancet Commission (31) and in the adapted PHD-2025 (13). Also, we explicitly included saturated fat intake as an additional component, which was not captured by Stubbendorff et al. (23) since no information about palm oil or lard was available in their study.

The PHD frameworks themselves have also been subject to ongoing scientific discussion. As modeled reference diets, they represent normative constructs per definition (9, 13). Critiques of the PHD-2019 framework have highlighted potential micronutrient shortfalls (55), as well as challenges related to affordability and applicability across diverse populations (56). Since the PHD-2025 was only recently published, its implications have not yet been evaluated to the same extent. However, recent analyses suggest that although the revised framework addresses some earlier concerns, important challenges remain, especially in lower-income settings (57). In addition, recent scientific discussions led by Zagmutti et al. in early 2026 have raised further methodological considerations, including the assumptions underlying mortality estimates, the extent to which uncertainties were addressed, and the transparency model (58). Further, the evidence base for intake recommendations and their implications for micronutrient adequacy remain under ongoing discussion across both PHD-2019 and PHD-2025 (55–58).

In this study, the average PHDI-2019 was 19.3 (median = 19.0) out of 42 possible points, corresponding to approx. 46.0% of the maximum score or a moderate adherence to the PHD. The updated PHDI-2025 yielded very similar results, with a mean of 20.6 out of 45 points (45.8%), indicating that the 2025 revisions, particularly the inclusion of sodium, did not substantially change the overall adherence distribution. Internationally, similar adherence levels have been reported. For PHD-2019, Stubbendorff et al. (23) described a mean score of 17.9 points out of 42 (approx. 43%) in the Swedish Malmö Diet and Cancer cohort (*n* = 22,421, 45–73 years), recruited between 1991 and 1996. Cacau et al. (15) reported a mean of 60.4 out of 150 points (approx. 40%) for Brazilians (2008–2010, *n* = 14,779), while Frank et al. (16) found a median PHDI-2019 of 66.9 of 140 possible points (approx. 48%) in the U. S. NHANES (2003–2018, *n* = 33,859). For Germany, the most recent nationally representative data were derived from German Health Interview and Examination Survey for Adults (DEGS1, 2008–2011, *n* = 7,987), with a reported mean PHDI-2019 of 39% (22). Although these studies have limited methodological comparability, they all suggest that adherence to PHD is moderate and broadly similar in these high- and middle-income populations, with substantial room for improvement.

### 4.4 Agreement

The agreement analysis between the mHEI-2015 and the two versions of PHDI was statistically significant. However, a weighted kappa of 0.34 for PHDI-2019 and 0.38 for PHDI-2025 indicates fair agreement based on the classification by Fleiss et al. (42), while a Pearson’s correlation coefficient of 0.52 and 0.58, respectively, demonstrated a moderate linear association. These findings suggest that, although both frameworks are grounded in health-promoting dietary principles, they capture distinct dimensions of diet quality. This is consistent with previous research. For example, Frank et al. (17) reported a Pearson’s correlation of 0.65 between HEI-2015 and PHDI-2019 in U. S. NHANES data (2015–2018, *n* = 8,128). Similarly, Richter et al. (22) reported a Pearson’s correlation of 0.6 in DEGS1. While numerically higher, such coefficients still fall within the range of a moderate correlation (59). Richter et al. further demonstrated that 7% of participants in the highest HEI-2015 quartile fall into the lowest PHDI-2019 quartile, highlighting the incomplete overlap between nutritional and environmental diet quality dimensions (22).

While the HEI, including HEI-2005, HEI-2010, and HEI-2015, has been repeatedly associated with favorable health outcomes, including lower risks of all-cause mortality and major chronic diseases (6–8), it remains debatable whether specific components, such as total protein foods or dairy, are either the primary drivers of these associations or sufficiently specific. The PHDIs, despite applying stricter limits on animal-source foods due to environmental concerns, have likewise been linked to lower risks of cardiovascular disease, type 2 diabetes, all-cause mortality, and others (17, 21, 23). Despite different compositional strategies, both frameworks lead to similar health-promoting outcomes. This suggests that both indices converge on core dietary principles, i.e., high intake of plant-based foods, low added sugar, and a favorable fat profile, which are likely to drive these health benefits.

The recently updated PHD-2025 reflects these same core dietary principles while refining the balance between health and environmental considerations (13). Compared to the 2019 framework (9), the revised PHD modestly increased the reference ranges for several animal-sourced foods, particularly meat, eggs, and fish, and incorporated sodium as a new health-related component. These adjustments were informed by an expanded evidence base, including new meta-analyses and long-term cohort data linking modest consumption of nutrient-dense animal products to improved micronutrient adequacy, especially for calcium, vitamin B12, iron, and iodine, without substantially compromising environmental sustainability when kept within planetary boundaries.

### 4.5 Environmental impacts

In the present study, WFP was derived from the SuEATableLife database, whereas GHGE and LU were derived from SHARP-ID. As these databases differ in their system boundaries, particularly with regard to post-retail stages (33, 34), the resulting indicators are not directly equivalent. Therefore, the observed WFP metric should not be interpreted with the same inferential weight as the GHGE and LU data. A more detailed discussion of database-related methodological differences in the BVS III context has been published elsewhere (27).

The PHD was developed in 2019 explicitly to integrate health and environmental considerations (9). Accordingly, the observed reductions in GHGE and LU across increasing PHDI quintiles are consistent with its underlying framework. In contrast, the mHEI-2015 was not associated with lower GHGE and showed only modest reductions in LU. This is in line with U. S. NHANES data (2015–2018, *n* = 8,128). Frank et al. (18) showed that PHDI-2019 adherence resulted in twice the GHGE reductions compared to HEI-2015 (−0.4 against −0.2 kg CO_2_eq per SD, respectively). Similarly, modeled diets from the French NutriNet-Santé cohort (2014, *n* = 29,413) demonstrated that an optimized alignment with the PHD-2019 reduced environmental impacts substantially (up to 75% lower GHGE and 57% lower LU) by a 141% improvement in PHDI-2019, albeit with nutritional adjustments in the model for iron and zinc bioavailability constraints (20). In the new 2025 PHD version, micronutrient adequacy was, hence, improved (13). Conversely, the EHU12/24 study (2014–2017) with 603 Spanish university students aged 18–28 years found that diets with lower GHGE were more frequently consumed by individuals with lower HEI-2010 scores (*β* = 0.039 kg CO_2_eq/1,000 kcal/d), concluding that diets of the highest nutritional quality were not necessarily those with the lowest environmental impacts (24). This further highlights the limited alignment between health and sustainability metrics, reinforcing the need for integrated approaches such as the PHD (9, 13) and the recent revisions of national guidelines exemplified by Germany (60), Austria (61), Switzerland (62), Netherlands (63), and other countries (64–66).

Gebara et al. (67) showed that plant-oriented diets, particularly vegan, vegetarian, or flexitarian, can reduce climate impact by up to sevenfold while increasing healthy life expectancy. Only diets limiting total meat intake to a maximum of 255 g per week, primarily poultry and pork, met all modeled health and environmental constraints. These findings are in line with recent analyses of the BVS III dataset published elsewhere, which showed that vegetarian and vegan diets in Bavaria were associated with approximately 24 to 34% lower environmental impacts per 2,500 kcal compared to omnivorous diets (27), while maintaining nutritional adequacy (68). Together, these studies underscore not only the feasibility but also the particular responsibility of high-income countries, where the potential to reduce the environmental impacts is greatest and the ethical imperative for change is most pronounced.

## 5 Limitations

Several limitations should be considered when interpreting the results of this study. The overall response rate of 26.0% reflects broader trends of declining participation in population-based surveys. To address this, sampling strategies and weights were applied to improve representativeness relative to the Bavarian adult population. However, residual selection bias cannot be fully excluded. Particularly, participants may differ systematically from non-participants in characteristics relevant to survey participation and dietary behavior. As no additional information on non-participants was available beyond fieldwork documentation, a formal non-responder analysis was not feasible. Second, there is currently no internationally standardized approach for operationalizing the PHD. The PHDIs applied here were adapted from previously established methods and modified to align with the structure and level of detail of the BVS III dataset. The PHDI-2025 was extrapolated based on the existing framework. Although food processing represents a relevant dimension of dietary quality, the degree of processing was not explicitly assessed and is only indirectly reflected in the HEI and PHD through proxy components. The environmental impact estimates are dependent on the underlying life cycle assessment databases. As system boundaries and assumptions vary across databases, absolute values of GHGE, LU, and WFP may differ when alternative sources are applied. In addition, the databases differ in system boundaries. Particularly, WFP estimates terminate at the distribution center and do not include post-retail stages. Accordingly, WFP estimates are not equivalent regarding the inferential framework to GHGE and LU. Dietary assessment was based on interviewer-led but self-reported 24-h recalls, which are subject to recall bias and may be influenced by the structure of the dietary software and food composition database. Although multiple 24-h recalls reduce within-person variation, some degree of measurement error and potential misclassification cannot be excluded. The anthropometric variables body weight and height were self-reported and may therefore be subject to reporting bias, although predefined plausibility ranges and control prompts were implemented during data collection. Lastly, the BVS III was conducted during the COVID-19 pandemic, which introduced logistical constraints during recruitment and data collection.

## 6 Conclusion

This study assessed the alignment between the mHEI-2015 and two versions of the PHDI (PHDI-2019 and PHDI-2025), their sociodemographic associations and associated environmental impacts in a representative sample of Bavarian adults. While both index frameworks reflect aspects of health-promoting diets, agreement between them was limited, indicating that health-focused and planetary-oriented diet quality indices overlapped only partially. Female sex, higher education, and urban residence were common positive predictors of adherence to all indices, i.e., sociodemographic characteristics that have also been consistently associated with health-conscious dietary behaviors such as vegetarianism and veganism. In contrast, older age was positively associated with mHEI-2015, but not with any PHDI, suggesting that dietary adaptations in older individuals may prioritize conventional health aspects over environmental considerations. This study provides the first empirical evaluation of the recently adapted PHD-2025 framework through the newly developed PHDI-2025, offering timely evidence on how the 2025 updates affect health and environmental alignment, indicating that the revision refined rather than redefined the framework.

As part of the high-income country Germany, and itself a high-income federal state relative to the other German states, Bavaria bears a particular social and ethical responsibility to support dietary transitions that are both health-promoting and environmentally sustainable. The integration of sustainability criteria into the revised German FBDGs represents a critical step toward harmonizing ecological and nutritional goals. However, these FBDGs remain recommendations and must now be translated into actionable national guidelines. Bavaria’s nutritional strategy, subtitled “Healthy, sustainable, enjoyable, and regional”, appropriately references the PHD and sets ambitious targets, including increased promotion of legumes and nuts, while also constructively critiquing the national FBDGs. These goals are scientifically sound and align with both current evidence and societal expectations. The next decisive step is to ensure that these objectives are communicated clearly and compellingly to the population.

## Data Availability

The datasets presented in this article are not readily available because the data that support the findings of this study will be available following the publication of the primary results in the coming months. Afterward, they can be requested upon reasonable justification, including a description of the intended analysis and approval from the project partners. All requests must ensure proper use, with any misuse strictly prohibited. Requests to access the datasets should be directed to Kurt Gedrich, kgedrich@tum.de.
