# Supplementary material for: Alignment between healthy and sustainable diets: comparing the 2019 and 2025 adaptations of the planetary health diet with the healthy eating index in the Bavarian population
Source: Front Nutr. 2026 Apr 29;13:1740957. doi: 10.3389/fnut.2026.1740957 (PMC13167579; doi:10.3389/fnut.2026.1740957)
Supplement: Supplementary file 1 [file Table_1.docx]

Supplementary Material

# Supplementary Figures and Tables

## Supplementary Tables

**Supplementary Table 1 Sociodemographic characteristics, Planetary Health Diet Index-2019, Planetary Health Diet Index-2025, and mHEI-2015 of the unweighted study population (n = 1,100). Values are given as % (n), unless specified otherwise.**

| **Variable** | **n = 1,100** |
| --- | --- |
| **Sex** |  |
| Male | **44 (485)** |
| Female | **56 (615)** |
| **Mean (SD) age in years** | **49.3 (14.9)** |
| **Age group** |  |
| 18-24 | **6 (71)** |
| 25-34 | **14 (156)** |
| 35-50 | **27 (297)** |
| 51-64 | **35 (387)** |
| ≥65 | **17 (189)** |
| **Mean (SD) BMI in kg/m^2^** | **25.8 (4.8)** |
| **BMI group** |  |
| Underweight | **2 (23)** |
| Normal weight | **47 (520)** |
| Pre-obesity | **33 (367)** |
| Obesity | **17 (190)** |
| **Education** |  |
| Low | **20 (220)** |
| Medium | **29 (317)** |
| High | **51 (563)** |
| **Smoking status** |  |
| Never | **53 (587)** |
| Currently | **16 (172)** |
| In the past | **31 (340)** |
| **Physical activity group** |  |
| Sedentary | **27 (294)** |
| Low active | **27 (298)** |
| Active | **23 (250)** |
| Very active | **23 (258)** |
| **Mean (Median; IQR) Planetary Health Diet Index-2019** | **19.6 (19.0; 16.0, 23.0)** |
| **Mean (Median; IQR) Planetary Health Diet Index-2025** | **21.1 (21.0; 18.0, 24.0)** |
| **Mean (Median; IQR) mHEI-2015** | **52.5 (52.0; 43.9, 60.9)** |
| Abbreviations: BMI body mass index, IQR interquartile range, mHEI metric Healthy Eating Index, SD standard deviation | |

**Supplementary Table 2 Diet-related environmental sustainability metrics across PHDI and mHEI-2015 quintiles in Bavaria. Sustainability metrics are adjusted to an energy intake of 2,500 kcal. WFP was estimated using a database with system boundaries different from those used for GHGE and LU; direct comparison across indicators should therefore be interpreted cautiously. Data are weighted to represent the Bavarian population.**

| **Planetary Health Diet Index-2019 quintiles** | | | | | | | |
| --- | --- | --- | --- | --- | --- | --- | --- |
| **Characteristic** | **Lowest, n = 304 (28%)*^1^*** | **Low, n = 189 (17%)*^1^*** | **Medium, n = 220 (20%)*^1^*** | **High, n = 208 (19%)*^1^*** | **Highest, n = 179 (16%)*^1^*** | **p-value*^2^*** | **p-trend*^3^*** |
| **GHGE (in kg CO_2_eq)** | 6.4 (6.1; 5.3, 7.3) | 6.2 (6.1; 5.2, 6.8) | 6.3 (6.2; 5.4, 7.1) | 6.2 (5.9; 5.1, 7.0) | 5.4 (5.2; 4.3, 6.3) | <.001 | <.001 |
| **LU (in m^2^*yr)** | 8.2 (7.8; 6.7, 9.5) | 7.8 (7.4; 6.0, 8.7) | 7.8 (7.4; 6.4, 8.6) | 7.4 (6.7; 5.6, 8.7) | 5.8 (5.3; 4.5, 6.6) | <.001 | <.001 |
| **WFP (in kiloliters)** | 4.3 (4.1; 3.6, 4.8) | 4.3 (4.2; 3.6, 5.1) | 4.5 (4.0; 3.6, 5.3) | 4.5 (4.1; 3.7, 5.1) | 4.4 (4.1; 3.4, 4.8) | .828 | .305 |
| **Planetary Health Diet Index-2025 quintiles** | | | | | | | |
| **Characteristic** | **Lowest, n = 280 (25%)*^1^*** | **Low, n = 219 (20%)*^1^*** | **Medium, n = 170 (15%)*^1^*** | **High, n = 226 (21%)*^1^*** | **Highest, n = 205 (19%)*^1^*** | **p-value*^2^*** | **p-trend*^3^*** |
| **GHGE (in kg CO_2_eq)** | 6.4 (6.3; 5.3, 7.3) | 6.1 (6.0; 5.2, 6.8) | 6.4 (6.2; 5.4, 7.3) | 6.1 (5.7; 5.1, 7.0) | 5.6 (5.4; 4.4, 6.6) | .002 | <.001 |
| **LU (in m^2^*yr)** | 8.4 (8.1; 7.2, 9.6) | 7.7 (7.4; 6.3, 8.6) | 7.9 (7.4; 6.3, 8.6) | 7.2 (6.8; 5.6, 8.4) | 6.0 (5.3; 4.6, 6.7) | <.001 | <.001 |
| **WFP (in kiloliters)** | 4.3 (4.1; 3.6, 4.7) | 4.3 (4.0; 3.6, 5.1) | 4.4 (4.1; 3.5, 4.9) | 4.5 (4.1; 3.7, 5.1) | 4.5 (4.1; 3.5, 5.0) | .842 | .146 |
| **mHEI-2015 quintiles** | | | | | | | |
| **Characteristic** | **Lowest, n = 220 (20%)*^1^*** | **Low, n = 221 (20%)*^1^*** | **Medium, n = 220 (20%)*^1^*** | **High, n = 219 (20%)*^1^*** | **Highest, n = 220 (20%)*^1^*** | **p-value*^2^*** | **p-trend*^3^*** |
| **GHGE (in kg CO_2_eq)** | 6.1 (5.7; 5.2, 6.8) | 6.0 (5.8; 5.0, 6.6) | 6.3 (6.3; 5.1, 7.4) | 6.4 (6.2; 5.5, 7.2) | 5.9 (5.7; 4.6, 7.0) | .063 | .849 |
| **LU (in m^2^*yr)** | 8.0 (7.5; 6.6, 8.6) | 7.6 (7.3; 6.1, 8.7) | 7.7 (7.4; 6.0, 8.8) | 7.5 (7.2; 5.9, 8.7) | 6.7 (6.2; 4.9, 7.8) | .004 | .008 |
| **WFP (in kiloliters)** | 4.2 (4.0; 3.6, 4.8) | 4.1 (4.0; 3.5, 4.5) | 4.4 (4.0; 3.6, 5.2) | 4.4 (4.2; 3.7, 4.8) | 4.9 (4.5; 3.9, 5.5) | .002 | .001 |
| Abbreviations: CO2eq carbon dioxide equivalents, GHGE greenhouse gas emissions, IQR interquartile range, LU land use, mHEI metric Healthy Eating Index, WFP water footprint | | | | | | | |
| *^1^*Mean (Median; IQR) | | | | | | | |
| *^2^*Design-based Kruskal Wallis test | | | | | | | |
| *^3^*Design-based generalized linear model with ordered factor levels | | | | | | | |

## Supplementary Figures


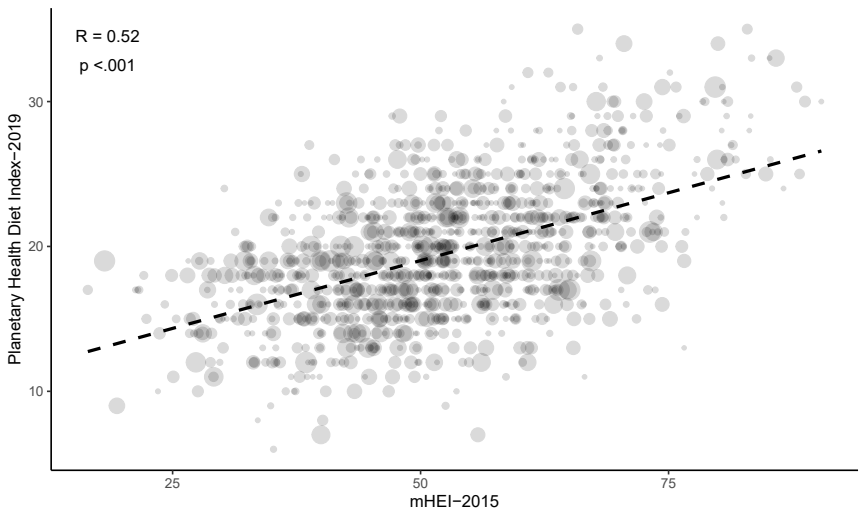


Supplementary Figure 1 Weighted correlation between the Planetary Health Diet Index‑2019 and mHEI-2015 (*n* = 1,100). The bubble size represents the survey weight of the observation. Data are weighted to represent the Bavarian population.
Abbreviations: mHEI metric Healthy Eating Index,


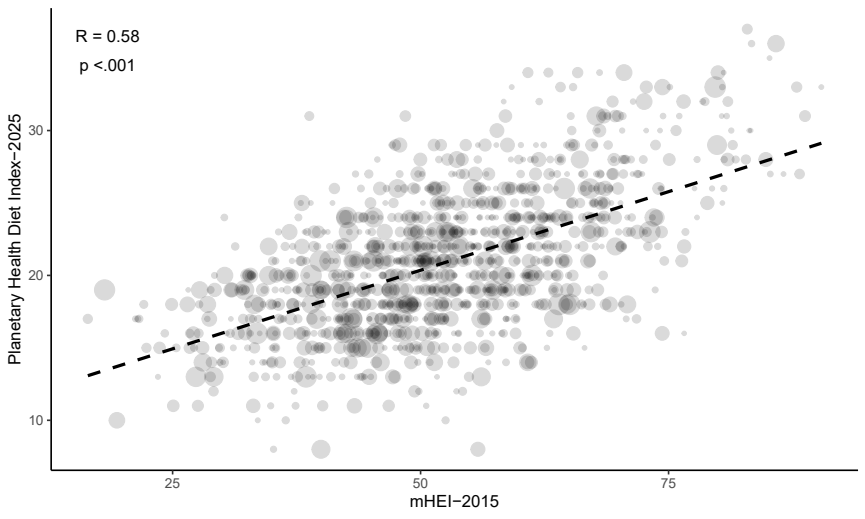


Supplementary Figure 2 Weighted correlation between the Planetary Health Diet Index‑2025 and mHEI-2015 (*n* = 1,100). The bubble size represents the survey weight of the observation. Data are weighted to represent the Bavarian population.
Abbreviations: mHEI metric Healthy Eating Index,


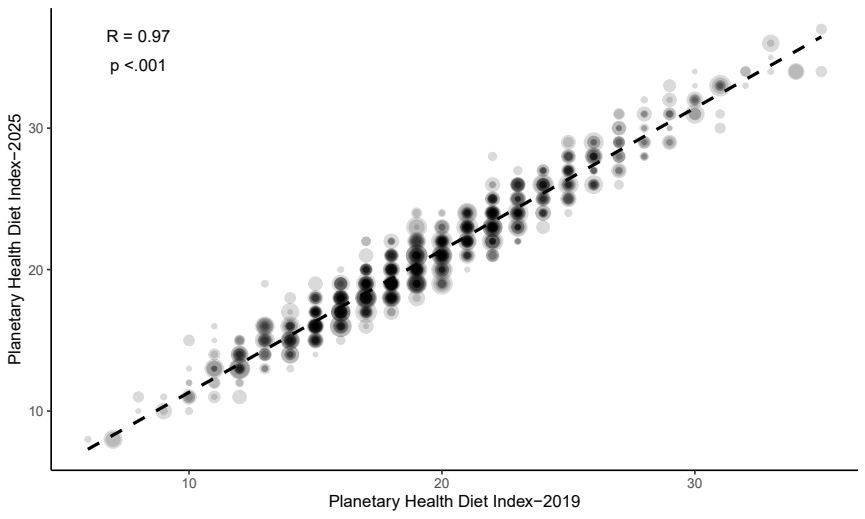


Supplementary Figure 3 Weighted correlation between the Planetary Health Diet Index‑2025 and the Planetary Health Diet Index‑2019 (*n* = 1,100). The bubble size represents the survey weight of the observation. Data are weighted to represent the Bavarian population.


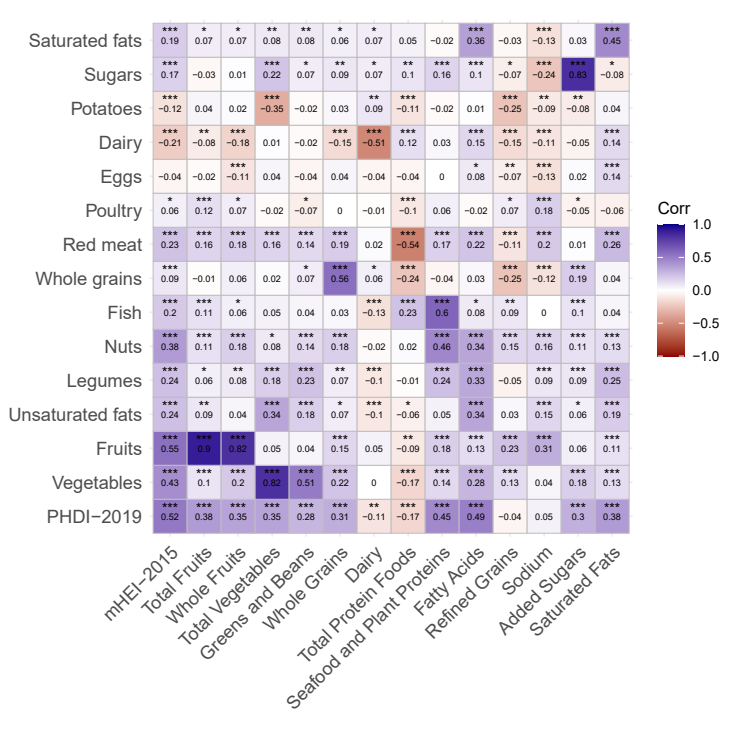


Supplementary Figure 4 Pearson’s correlations between the Planetary Health Diet Index-2019, including its components (y-axis), and mHEI-2015 and its components (x-axis) (n = 1,100). Red indicates negative and blue positive correlation. No correlation is represented as white. Data are weighted to represent the Bavarian population. Significance levels: * *p*<.05, ** *p* <.01, *** *p* <.001).
Abbreviations: mHEI metric Healthy Eating Index, PHDI Planetary Health Diet Index


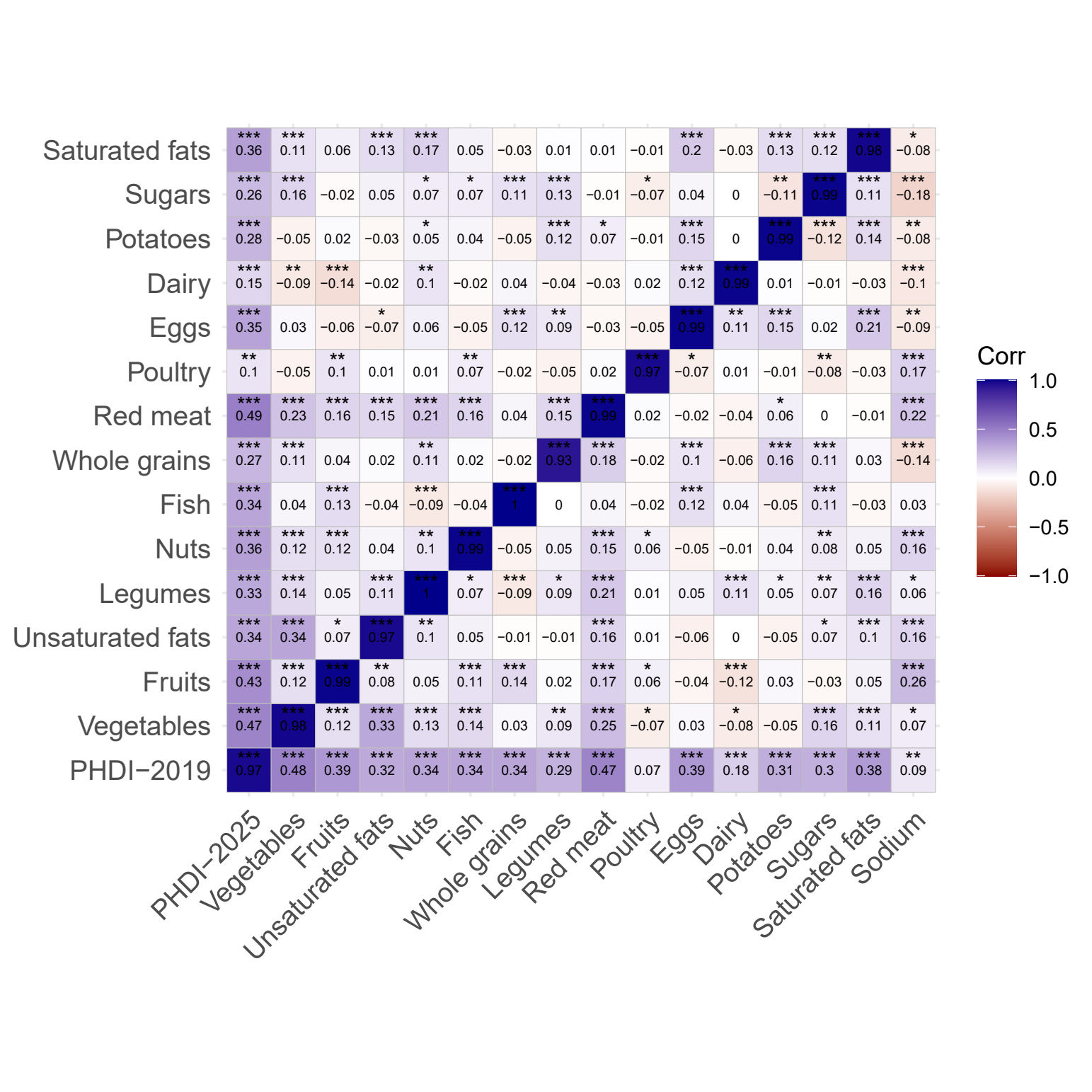


Supplementary Figure 5 Pearson’s correlations between the Planetary Health Diet Index-2025, including its components (y-axis), and Planetary Health Diet Index-2019 and its components (x-axis) (n = 1,100). Red indicates negative and blue positive correlation. No correlation is represented as white. Data are weighted to represent the Bavarian population. Significance levels: * *p*<.05, ** *p* <.01, *** *p* <.001).
Abbreviations: PHDI Planetary Health Diet Index


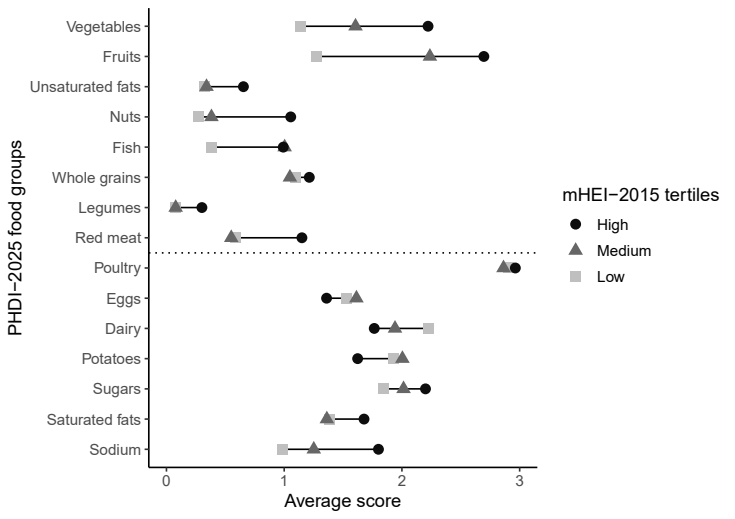


Supplementary Figure 6 Dotchart of the average scores across the PHDI-2025 food groups by mHEI-2015 (n = 1,100). Food groups for recommended consumption are shown above and for discouraged consumption below the dotted line. Data are weighted to represent the Bavarian population.
Abbreviations: mHEI metric Healthy Eating Index, PHDI Planetary Health Diet Index
